# The simultaneous occurrence of a fungal ball in maxillary and sphenoid sinuses: a case report and review of the literature

**DOI:** 10.1093/jscr/rjaf014

**Published:** 2025-02-28

**Authors:** Aishah A AlGhuneem, Jood K Alotaibi, Maha A AlHarbi, Ali H Almomen

**Affiliations:** Department of General Surgery, Royal Medical Services, Bahrain Defence Force Hospital, Wali Al Ahed Highway, PO Box 28743, West Riffa, Kingdom of Bahrain; College of Medicine, Imam Abdulrahman Bin Faisal University, King Faisal Ibn Abd Al Aziz, Dammam 34212, Eastern Province, Saudi Arabia; Department of Otolaryngology-Head and Neck Surgery, Ministry of Health, 28th street, Ghirnatah, Dammam 32245, Eastern Province, Saudi Arabia; ENT Department, King Fahad Specialist Hospital, Ammar Bin Thabit St., Al Merikbat district, Dammam 32253, Ministry of Health, Eastern Province, Saudi Arabia

**Keywords:** sinonasal fungal ball, fungal rhinosinusitis, functional endoscopic sinus surgery, maxillary sinus

## Abstract

A fungal ball is most frequently found in the maxillary sinus, though it can also occur in the sphenoid, ethmoid, and frontal sinuses. This study aims to demonstrate the pathological conditions and clinical features of a patient with unilateral left maxillary and sphenoid sinus fungal balls. A 52-year-old woman arrived with complaints of headache, facial pain, and post-nasal discharge. An endonasal endoscopic examination was unremarkable. However, the plain paranasal sinuses computerized tomography scan (CT scan PNS) of the patient revealed two simultaneous opacities occupying both the maxillary and sphenoid sinuses with calcification indicating a fungal ball occupying. Even with the advancing diagnostic methods and investigations, detecting a fungal ball remains difficult. A CT scan PNS and a nasal endoscopy with histopathological evaluation are considered useful and specific. Endonasal endoscopic sinus surgery is the gold standard, with a high success rate and low morbidity rate.

## Introduction

Over the past two decades, the identification of fungal rhinosinusitis has been easier with the advancement of clinical assessment tools, where a classification of fungal rhinosinusitis into invasive and non-invasive forms has been established. Fungal balls are identified as a form of non-invasive fungal rhinosinusitis characterized by the collection of fungal material within the paranasal sinus with a predilection to a unilateral involvement of the maxillary sinus [1]. The occurrence of a fungal ball in bilateral paranasal sinuses is rare, with limited cases reported in literature. The exact underlying pathogenesis of fungal balls remains unclear; however, the occurrence of a fungal ball is commonly seen in an immunocompetent elderly female with an average age of 64 [1]. These patients clinically present with a complaint of nasal obstruction, facial pain, post-nasal drip (PND), and purulent rhinorrhea [1]. In addition, a range from normal appearing mucosa and nasal cavity to a crusted, edematous, purulent nasal cavity with polyposis could be observed in the endoscopic examination [1]. Radiological imaging is needed to aid the diagnosis of a fungal ball; a computed tomography (CT) of the paranasal sinuses will show a classical finding of nasal sinus opacification with a characteristic intralesional hyperdensity or calcification [1]. Further histopathological evaluation will lead to the definitive diagnosis of a fungal ball showing a dense matter of fungal hyphae without invasive features [1]. In this paper, we present a case of a 52-year-old female with a simultaneous occurrence of a fungal ball in the maxillary and sphenoid sinuses.

## Case presentation

**Figure 1:**
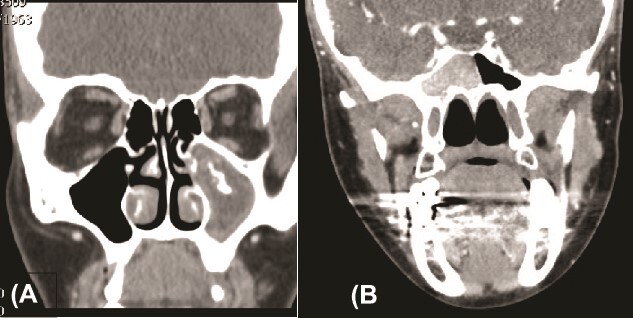
Paranasal sinuses CT scan, coronal view, showing left maxillary sinus fungal ball (A) and a right sphenoid fungal ball (B).

**Figure 2:**
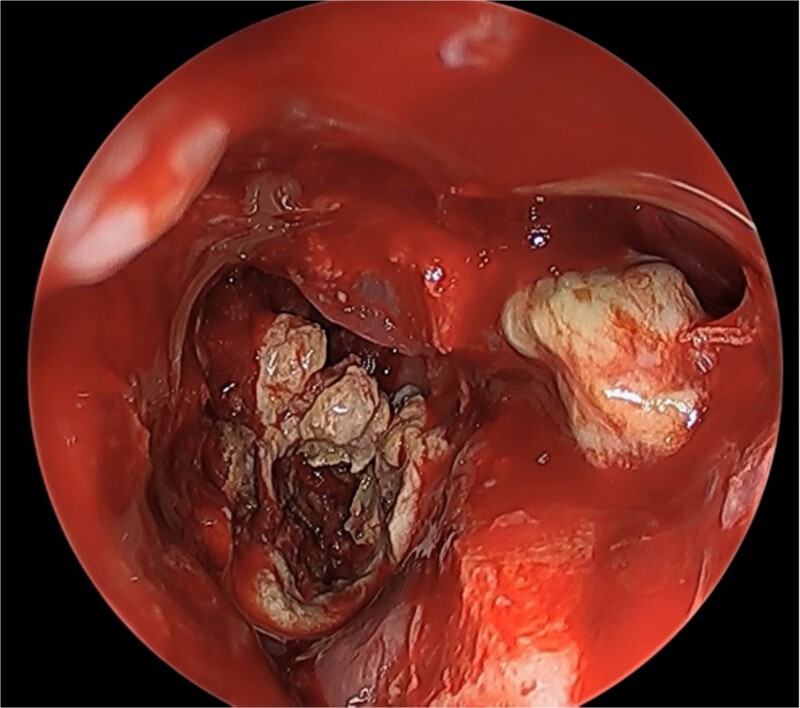
Endoscopic view of simultaneous occurrence of the fungal ball in maxillary and sphenoid sinuses.

**Figure 3:**
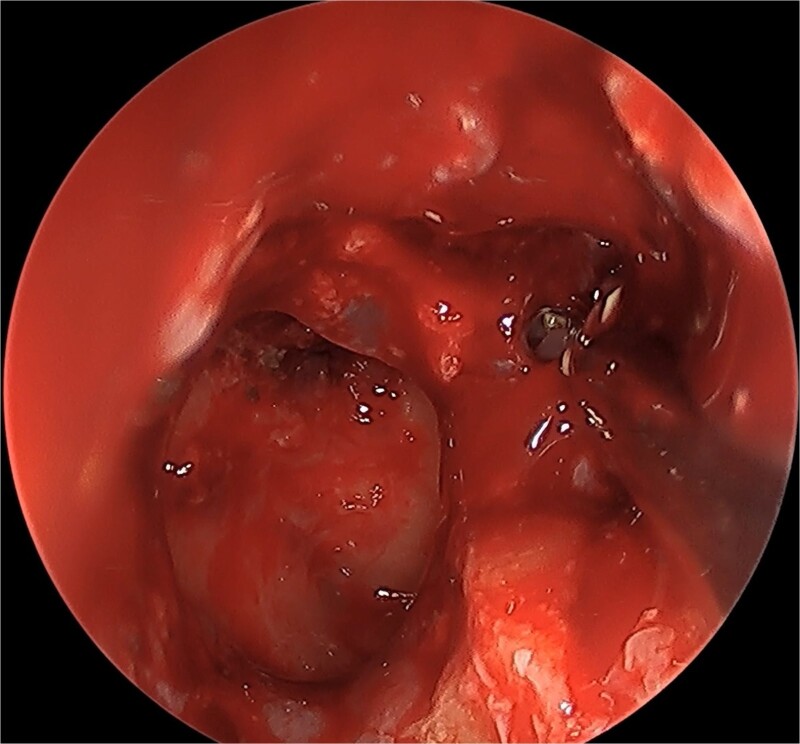
Post-operative endoscopic view post sphenoidectomy and maxillary antrostomy.

We present a case of a 52-year-old female, not known to have any chronic disease, who presented to our institution’s otorhinolaryngology clinic with a complaint of headache, facial pain, and PND for 6 months. Endonasal endoscopic examination was unremarkable. A paranasal sinus CT was requested to aid the diagnosis, which showed two simultaneous opacities occupying the left maxillary and right sphenoid sinuses, respectively (Fig. 1A and B). The opacified lesions were associated with calcifications, metallic shadowing, and bony thickening of the sinus wall. According to the history and radiological imaging findings, a diagnosis of a fungal ball occupying both the maxillary and sphenoid sinuses was achieved, and a functional endoscopic sinus surgery (FESS) with left wide maxillary antrostomy removal of the thick fungal debris occupying the left maxillary sinus (Fig. 2) was decided to be performed. In addition, a wide endoscopic sphenoidotomy was performed, and the sphenoid sinus was full of fungal debris (Fig. 3), which was cleaned thoroughly. The patient had an uneventful post-operative course.

## Discussion

Fungal rhinosinusitis is a relatively rare disease classified into invasive and non-invasive forms; these forms encompass both acute and chronic courses of illness [1]. The non-invasive form of fungal rhinosinusitis is further classified into four subcategories: the saprophytic form, allergic fungal sinusitis, eosinophilic fungal rhinosinusitis, and fungus ball [1]. The latter is considered the most common form of non-invasive rhinosinusitis, which is usually reported to cause unilateral disease with a predilection to the maxillary sinus [2]. Differentiating between allergic fungal rhinosinusitis and fungal balls is crucial, as allergic fungal rhinosinusitis typically appears widespread, affecting both sinuses and often accompanied by nasal polyps. On the other hand, fungal ball typically occurs on one side and predominantly affects the maxillary sinus [1].

The literature postulated that most cases (84%) of fungus balls involved the maxillary sinus, followed by the sphenoid sinus (10%), and less commonly, the ethmoid (2%) and a less frequent maxillary and anterior ethmoid sinuses (9%) involvement were also reported [3]. The rareness of bilateral fungal balls complicates the diagnosis of this disease. In 2022, a retrospective cohort study was conducted on 28 patients who had bilateral fungal balls [3]. The study found that out of these cases, six patients (21%) had simultaneous fungal balls in both the maxillary and sphenoid regions [2]. It concluded that patients with bilateral fungal ball occurrence had an average age of 65.1, which was higher compared with those with unilateral occurrence [2]. Additionally, the bilateral group had a higher proportion of female patients and more comorbidities [2].

The exact pathogenesis of fungal rhinosinusitis remains unclear. However, the most isolated causative organism is Aspergillus [2]. Deviated nasal septum, paradoxical curvature of the middle turbinate, and history of endodontic sealers were reported to have a strong association with the occurrence of unilateral fungus balls [1]. On the other hand hypertension and immunocompromised status were more prevalent in bilateral fungal balls compared with unilateral fungal ball [2]. In our case, the patient was middle-aged female with no reported comorbidities or risk factors.

The modality of choice to diagnose a fungus ball in a CT scan, showing a hyper-attenuated calcified lesion representing a heterogeneous lesion occupying the sinus [3]. However, magnetic resonance imaging can be used if bone erosion and orbital or brain extensions are suspected [3]. Histopathologically, fungal balls have a characteristic accumulation of hyphae with alternating zones of different densities ranging from high to low representing an onion-like skin [1].

Symptomatic patients with a complaint of nasal obstruction, PND, facial pain, and purulent rhinorrhea are indicated for surgical intervention. Additionally, the presence of a heterogenous opacification in the sinus is an indication as well. Patients with a fungus ball are managed surgically through FESS with the routine post-operative care [2].

## Conclusion

The simultaneous occurrence of fungal balls in the sphenoid and maxillary sinuses is considered extremely rare with very limited reported cases in literature. While the diagnosis of fungal balls can be challenging due to non-specific history and physical examination findings, radiological imaging plays a crucial role in aiding diagnosis, and definitive histopathological confirmation is achieved through surgical intervention.

## References

[1] Grosjean P, Weber R. Fungus balls of the paranasal sinuses: a review. Eur Arch Otorhinolaryngol 2007;264:461–70. 10.1007/s00405-007-0281-5. 17361410

[2] Kim JS, Kwon SH, Kim JS, et al. Bilateral paranasal sinus fungal balls: a retrospective cohort study in 28 patients over a 21-year period. Medicine 2022;101:2–7. 10.1097/MD.0000000000030174. PMC9387944 35984143

[3] Seo YJ, Kim J, Kim K, et al. Radiologic characteristics of sinonasal fungus ball: an analysis of 119 cases. Acta Radiol 2011;52:790–5. 10.1258/ar.2011.110021. 21525111

